# Agronomic performance and technological quality of sugarcane submitted to different poultry litter dosages

**DOI:** 10.1038/s41598-024-66340-2

**Published:** 2024-07-04

**Authors:** Willian Marques Pires, Marconi Batista Teixeira, Frederico Antônio Loureiro Soares, Wilker Alves Morais, Adriano Carvalho Costa, Luiz César Lopes Filho, Jadson Belem de Moura

**Affiliations:** 1 Graduate Program in Agricultural Sciences – Agronomy, Federal Institute Goiano, Rio Verde, Goiás Brazil; 2 Graduate Studies in Social, Technological and Environment Science, Evangelical University of Goiás, Anápolis, Goiás Brazil; 3 Sedmo - Soil Research Group, Ecology and Dynamics of Organic Matter, Evangelical College of Goianésia, Goianésia, Brazil

**Keywords:** *Saccharum officinarum*, Sugar, Organic fertilizer, Alcohol, Poultry litter, Plant sciences, Biofuels

## Abstract

Sugarcane is a central crop for sugar and ethanol production. Investing in sustainable practices can enhance productivity, technological quality, mitigate impacts, and contribute to a cleaner energy future. Among the factors that help increase the productivity of sugarcane, the physical, chemical and biological parameters of the soil are amongst the most important. The use of poultry litter has been an important alternative for soil improvement, as it acts as a soil conditioner. Therefore, this work aimed to verify the best doses of poultry litter for the vegetative, reproductive and technological components of sugarcane. The experiment was carried out at Usina Denusa Destilaria Nova União S/A in the municipality of Jandaia, GO. The experimental design used was a complete randomized block design with four replications: 5 × 4, totaling 20 experimental units. The evaluated factor consisted of four doses of poultry litter plus the control (0 (control), 2, 4, 6 and 8 t ha^−1^). In this study, were evaluated the number of tillers, lower stem diameter, average stem diameter, upper stem diameter, plant height, stem weight and productivity. The technological variables of total recoverable sugar, recoverable sugar, Brix, fiber, purity and percentage of oligosaccharides were also evaluated. It was observed, within the conditions of this experiment, that the insertion of poultry litter did not interfere significantly in most biometric, productive and technological variables of the sugarcane. But it can also be inferred that there was a statistical trend toward better results when the sugarcane was cultivated with 4 t ha^-1^ of poultry litter.

## Introduction

Driven by the progress of the sugar and alcohol sector, to meet the high demand for sugar, mainly alcohol, in the national and international markets, the Brazilian Midwest region underwent a process of expansion of the area of sugarcane cultivation^1^.

In the 2011/12 harvest, the Midwest region had a cultivation area of 1,379.4 thousand ha, with a production of 95,566.2 thousand t, which corresponds to a productivity of 69,282.0 kg ha^−1^. For the 2021/22 harvest, the cultivated area was 1,806.7 thousand ha, for a production of 131,370.3 thousand t, which corresponded to a productivity of 72,712.0 kg ha^−1^. These values corresponded to increases of 31, 37 and 5% for cultivation area, production and productivity, respectively, in relation to the 2011/12 and 2021/22 harvests^1^.

The state of Goiás, in the 2011/12 harvest, was responsible for a cultivation area of 678.4 thousand ha, with a production of 48,032.1 thousand t, which corresponded to a productivity of 70,800.0 kg ha^−1^. For the 2021/22 harvest, the state had a cultivated area of 962.9 thousand ha, with a production of 71,898.3 thousand t; with this, a productivity of 74,672.0 kg ha^−1^ was obtained. These values corresponded to increases of 42, 50 and 5% for cultivation area, production and productivity, respectively, in relation to the 2011/12 and 2021/22 harvests^1^.

In view of the aforementioned information, it is observed that the need to increase productivity will be of great importance to satisfy the domestic and foreign markets. In this sense, among the factors that help increase the productivity of sugarcane, the physical, chemical and biological parameters of the soil are amongst the most important^2^.

The use of poultry litter has been an important alternative for soil improvement, as there is a large poultry market in the region, and this byproduct acts as a soil conditioner. These residues have considerable amounts of organic matter and nutrients that can be reused by plants^3^.

With the expansion of poultry production in Brazil, there was an increase in the supply of animal waste, such as poultry litter, and the consequent need to reuse or eliminate them. Thus, the use of this residue by the agricultural sector as an alternative source of nutrients has gained considerable importance in recent years^3^.

Silva^4^ reiterates that for the correct recommendation of organic residues, it is necessary to observe the cost of the product and the availability close to the place of application, since several factors, such as shipping, lack of knowledge of management methods, and even nutritional needs of the implanted culture, can be factors that determine the success or failure of its use.

Considering that according to IBGE data^5^ in the municipality of Rio Verde, more than 13 million heads of chickens were produced and considering the large generation of waste due to such intense production, the availability of poultry litter as an organic material is not a problem for the region and adjacent regions.

Because of the ability of organic matter to provide the soil with an increase in biological function, with an increase not only in microbial activity but also in the stability of soil aggregates, its addition to a productive system gives most crops an increase in production and productivity, which makes the use of poultry litter in sugarcane crops a valuable and promising option for recycling nutrients, improving soil quality and providing a more appropriate way of disposing of these residues^4^.

Therefore, this work aimed to verify the best doses of poultry litter in the vegetative, reproductive and technological components of sugarcane.

## Materials and methods

### Location of the experiment

**Figure 1:**
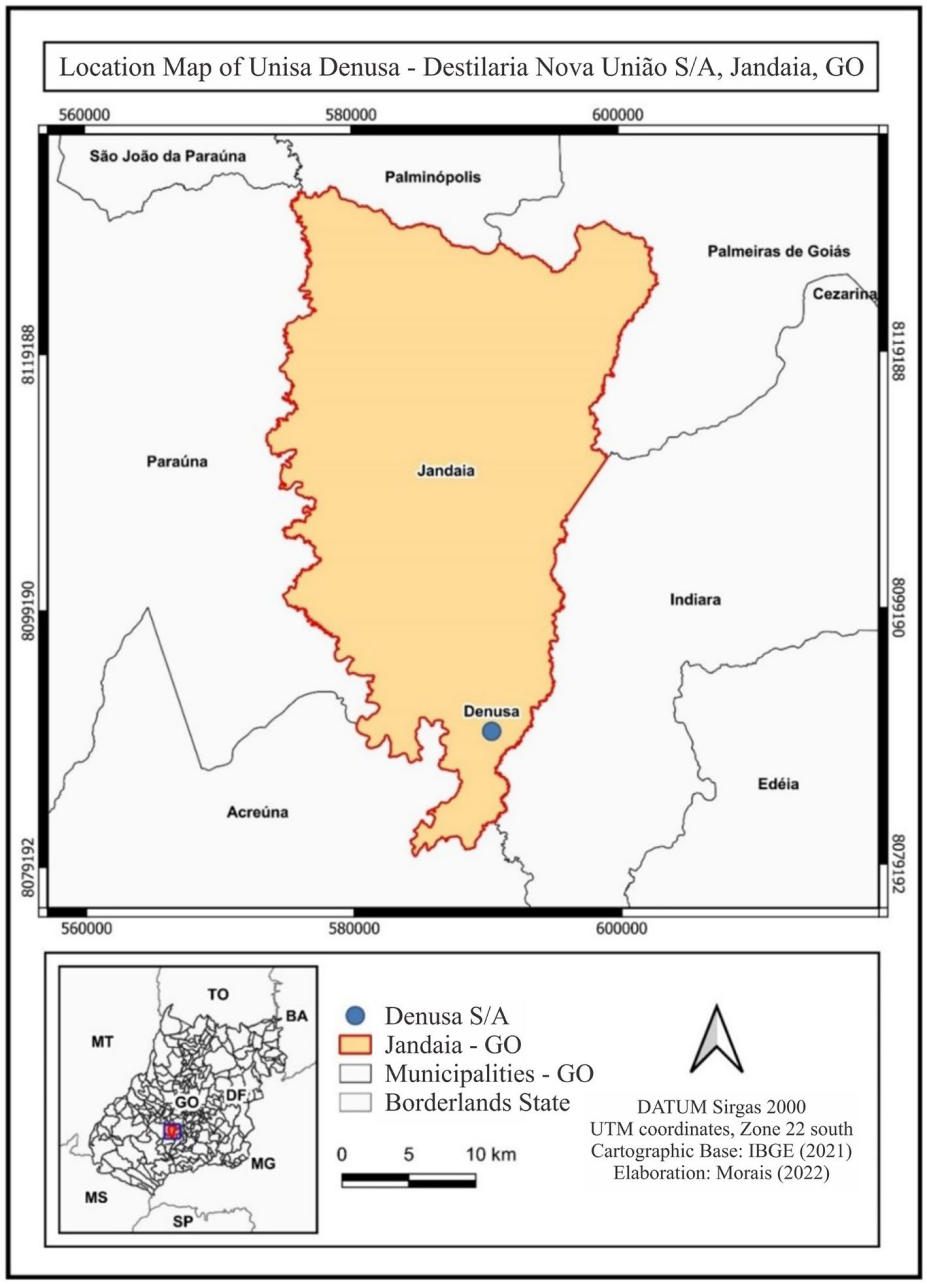
Location map of Denusa Sugar and Alcohol Plant—Destilaria Nova União S/A, in the municipality of Jandaia, Goiás, Brazil.

The experiment was carried out under field conditions in an area of the Braveza farm, belonging to Usina Denusa Destilaria Nova União S/A, in the municipality of Jandaia, GO. The geographic coordinates of the site are 17°16′34.53"S and 50°9′0.36"W (Fig. 1), with an average altitude of 650 m.

### Climate characteristics of the region

**Figure 2:**
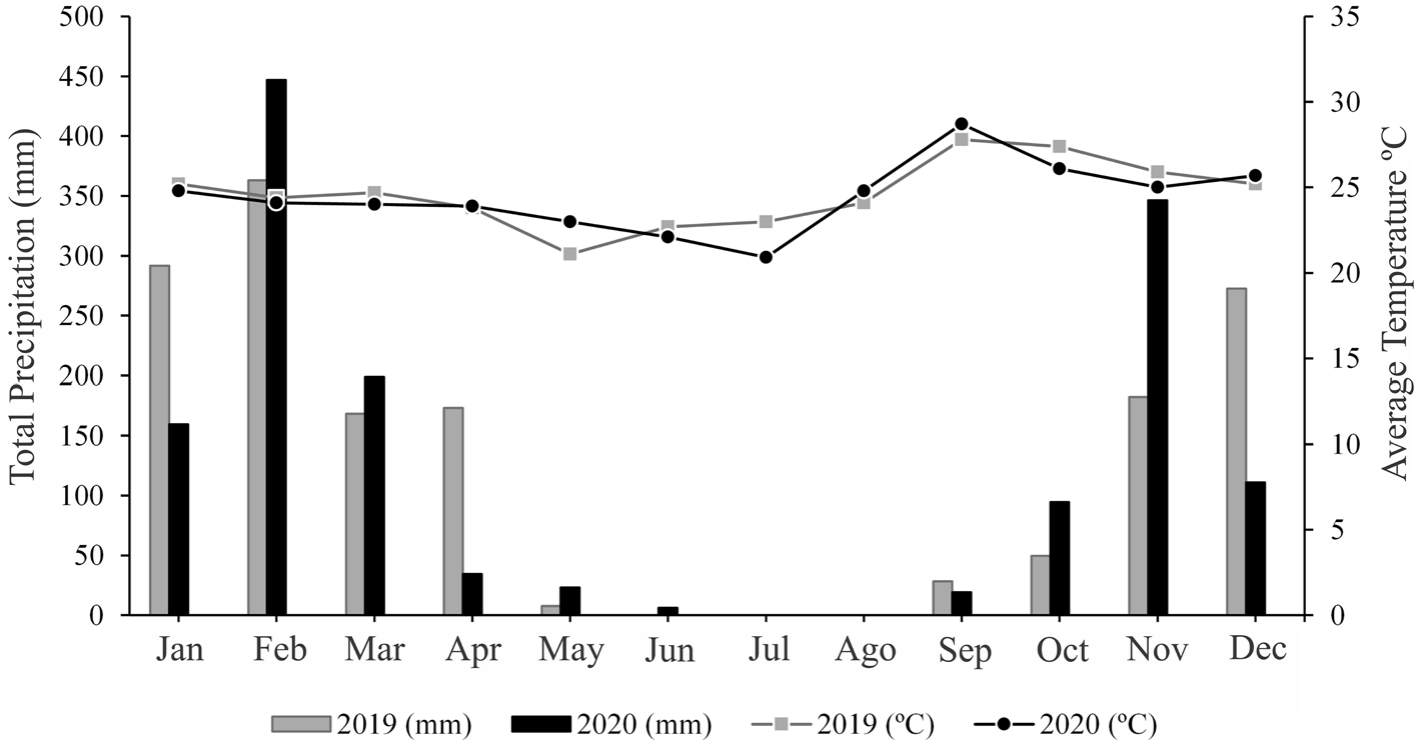
Total precipitation and monthly maximum temperature for 2019 and 2020 in the microregion of Vale do Rio dos Bois, Goiás, Brazil. *Source*: INMET, 2022.

According to the classification of Köppen and Geiger^5^, the climate of the region is type Aw, tropical, with rain in the months of October to April and dry in the months of May to September. The average temperature oscillates between 20 and 28 °C (in winter, there are occurrences of up to 5 °C). Annual precipitation reaches approximately 1,800 mm but is poorly distributed throughout the year, according to the climate data shown in Fig. 2.

### Characteristics of the experimental area

The soil in the experimental area is classified as an Eutrophic Red Latosol^6^ with a clayey texture, and the topography is flat. The chemical, physico-hydric, granulometry and textural classification of the samples collected before the installation of the experiment are described in Table 1.

**Table 1:** Chemical, physico-hydric, granulometry and textural classification of the soil in the experimental area, in layers of 0.0 and 0.20 m depth, Jandaia—GO, 2019/20 harvest.

Layer	pH	P _resin_	K ^+^	Ca ^2+^	Mg ^2+^	Al ^3+^	
m	CaCl_2_	mg dm^−3^	–––––––cmolc dm^−3^––––––––				
0.0–0.2	5.25	1.60	0.17	4.14	0.87	0.00	

Layer	H + Al	CEC	BS	Cu	Fe	Mn	Zn
m	––cmolc dm^−3^ ––		%	–––––––– mg dm ^-3^ ––––––––			
0.0–0.2	2.32	7.50	68.23	3.60	12.64	5.95	0.81

Layer	Granulometry (g kg^−1^)			Texture classification			
m	Sand	silt	Clay				
0.0–0.2	–	–	442.6	very clayey			

Chemical analysis manual for fertility assessment of tropical soils (IAC, 2001). CEC—Cation-exchange capacity; BS—CEC base saturation.

### Experimental design

The experimental design used was a randomized complete block design with four replications, namely, 5 × 4, totaling 20 experimental units. The evaluated factor consisted of four doses of poultry litter plus the control (0 (control), 2, 4, 6 and 8 t ha^−1^).

Each plot had 10 rows of sugarcane separated by 1.5 m (between rows), and each row was 10 m long, resulting in 3000 m^2^ of useful area. Therefore, each experimental plot was 150 m^2^. The six internal lines were used for evaluations. Additionally, between plots, there were borders of 4 m. The lines between the crops were 3 m wide.

Before the implementation of the experiment in the area, there was confinement. Soil preparation was carried out with subsoiling, plowing and harrowing at a depth of 0.40 m three months before the setting up of the experiment. Subsequently, limestone and gypsum were applied according to soil analysis.

The experiment was conducted in a first-year sugarcane, with planting in June 2019 and the first harvest in July 2020.

### Experiment implementation characteristics

Planting was mechanized with furrows 0.25 m deep, and the variety chosen to be cultivated in the experiment was IACSP95-5094.

This genotype has a medium cycle and was selected due to its high performance in the edaphoclimatic conditions of the region, its erect height, excellent sprouting of ratoons and adaptation to mechanization.

Cultural treatments referring to the use of herbicides, insecticides, fungicides and other products related to the control of invasive plants, pests and diseases were used according to the need and evaluation of infestation.

For irrigation, a total depth of 60 mm was applied after plant emergence, and subsequent irrigations were performed as needed. The irrigation method used was sprinkler, self-propelled reel type.

### Organic and mineral fertilization

The fertilization of the poultry litter was carried out according to the treatments. At the time of planting, fertilizer was applied manually on the side of the line (0.20 m) against the direction of the slope of the land.

The poultry litter came from a chicken farm in the municipality of Palmeiras de Goiás, GO, from the first batch of chickens, tanned, with the following chemical composition (average of applications) (Table 2).

**Table 2:** Chemical characteristics of poultry litter used in the sugarcane test, Jandaia—GO, 2019/20 harvest.

OM	pH	N	P	K	Ca	Mg	S
%		–––––––––––––––––––%–––––––––––––––––––					
67.32	7.20	2.54	0.74	0.85	7.58	0.91	1.52

OC	MM	C/N	Zn	Fe	Mn	Cu	B
–––––%–––––			––––––––––––––g kg^−1^––––––––––––––				
41.30	32.69	16.26	0.28	2.50	0.52	0.34	0.03

Humidity: 17.36% at 105 °C; MO: organic matter; OC: organic carbon; MM: mineral matter; C/N: carbon/nitrogen ratio.

All treatments were fertilized in the planting furrow with phosphorus (100 kg ha ^-1^) in the form of triple superphosphate (P_2_O_5_) and potassium (200 kg ha^−1^) in the form of K _2_ O and micronutrients, according to soil analysis results and recommendations by Raij et al.^7^ and Sousa and Lobato^8^.

### Biometric analysis

In the six central rows of the plots and subplots, the number of tillers (TILLERS), lower stem diameter (LSD), medium stem diameter (MSD), upper stem diameter (USD), plant height (PLH), weight stem (SW) and productivity (PROD) were measured. TILLERS was measured by counting tillers per line; the DC was determined with the aid of a caliper at the base of the plant and expressed in mm; the PLH was measured with the aid of a tape measure from the ground to the collar of the leaf + 1 (leaf + 1 is the one where the collar can be seen completely) and expressed in m.

### Productivity analysis

At harvest, SW was determined by weighing the total stalks present in the respective subplots, quantifying the weight of stalks in 10 m of the six central rows. Then, the value was extrapolated to t ha^−1^, aiming to assess the PROD of sugarcane. For this purpose, the cut was carried out as close to the ground as possible. The culms were then husked, and the pointer was detached. They were then weighed on a hook-type digital scale, *Soil Control brand* (accuracy = 0.02 kg), with a capacity of 50 kg.

### Technological analyses

Samples were collected from 10 stalks per treatment, which were submitted for technological analysis at the Agroindustrial Laboratory of Usina Denusa, in Jandaia—GO, to obtain total recoverable sugar (TRS), recoverable sugar (RS), Brix, fiber, purity and percentage of oligosaccharides (POL), according to the Consecana system^9^. To determine the quality of the technological attributes of sugarcane, the samples were disintegrated or crushed and homogenized. Then, 500 g of sample was taken and pressed in a hydraulic press for one minute at 250 kgf cm^−2^, resulting in two fractions: the juice and the wet bagasse (wet cake).

Quality indicators and recommended values for sugarcane are highlighted in Table 3.

**Table 3:** Quality indicators and recommended values for sugarcane.

Indicators	Recommended values	Source
TRS (sucrose, glucose, fructose)	> 15% (highest possible)	Ripoli and Ripoli (2004)
RS (glucose, fructose)	> 0.8%	Ripoli and Ripoli (2004)
		Consecana (2015)
BRIX	> 18%	Consecana (2015)
		Goncalves et al. (2021)
FIBER	11 to 13%	Ripoli and Ripoli (2004)
	12 ± 2%	Matsuoka (2017)
PURITY (POL/BRIX)	> 85%	Ripoli and Ripoli (2004)
		Consecana (2015)
POL	> 14	Ripoli and Ripoli (2004)
		Consecana (2015)

### Statistical analyses

The data were initially subjected to analysis of residues and variance considering the effects of treatment. Subsequently, analysis of variance was performed, and when significant, the adjustment of the linear models was verified (1st and 2nd degree). For models with 70% adjustment, the Skott–Knott test was performed.

Pearson’s correlation analysis was also performed (low: |r ≤ 0.30|; moderate: |0.30 ˂ r ≤ 0.70|; and high |r ˃ 0.70|).

Factorial multivariate analysis methods were performed from the average of the results of the four repetitions of each treatment for each variable. Standardization was performed using the following formula (observed value—mean)/deviation.

The computational software R version 4.2.2. and the Mvar.pt, ExpDes.pt and corrplot packages were used to perform all the analyses and generate the graphs^10–12^.

## Results

The analysis of variance showed a significant effect only for the variable tiller (Table 4), with the coefficient of variation being below 10% for most variables.

**Table 4:** Mean and standard error of the variables of sugarcane submitted to different doses of poultry litter.

PLD (t ha ^-1^ )	Biometry					
	TILLERING	FAULTS	LSD (mm)	MSD (mm)	USD (mm)	Height (cm)
0	13.25 ± 0.25 ^b^	12.41 ± 1.47	30.57 ± 1.13	27.53 ± 1.63	23.82 ± 1.21	326.00 ± 26.67
2	14.75 ± 0.25 ^a^	6.72 ± 1.54	29.16 ± 1.67	26.88 ± 0.99	21.73 ± 1.82	309.25 ± 21.54
4	15.00 ± 0.40 ^ab^	10.56 ± 2.28	29.32 ± 1.20	26.39 ± 1.13	21.45 ± 2.80	313.75 ± 18.20
6	13.50 ± 0.28 ^b^	9.73 ± 4.94	29.66 ± 2.01	25.46 ± 1.61	21.44 ± 1.29	319.75 ± 25.83
8	14.25 ± 0.25 ^a^	14.39 ± 3.29	30.45 ± 0.85	27.89 ± 1.00	21.70 ± 1.47	323.00 ± 17.56
CV (%)	4.42	29.69	2.69	3.80	7.55	6.82

	Production					
	AWS (kg)	AWL (kg)			PROD (t ha^−1^)	
0	1.93 ± 0.21	263.33 ± 41.65			175.64 ± 27.78	
2	1.89 ± 0.13	265.83 ± 50.59			177.31 ± 33.74	
4	1.91 ± 0.31	248.33 ± 53.35			165.64 ± 35.58	
6	2.05 ± 0.18	258.66 ± 47.10			172.53 ± 31.41	
8	2.00 ± 0.17	253.75 ± 22.54			169.25 ± 15.03	
CV (%)	6.93	15.14			15.14	

	Technological analysis					
	TRS (kg t^−1^)	RSS (%)	BRIX (%)	FIBER (%)	PURITY (%)	POL (%)
0	129.62 ± 7.35	0.62 ± 0.05	17.87 ± 0.69	10.95 ± 0.50	84.97 ± 2.02	12.86 ± 0.80
2	135.03 ± 6.01	0.60 ± 0.05	18.60 ± 0.58	11.10 ± 1.30	85.56 ± 1.99	13.44 ± 0.67
4	138.54 ± 8.86	0.57 ± 0.04	18.72 ± 0.98	10.45 ± 0.49	86.80 ± 1.74	13.83 ± 0.96
6	136.15 ± 3.78	0.62 ± 0.03	18.60 ± 0.49	10.27 ± 0.45	85.46 ± 1.31	13.55 ± 0.42
8	127.88 ± 9.00	0.66 ± 0.05	17.65 ± 1.07	10.32 ± 0.17	84.09 ± 1.70	12.65 ± 0.97
CV (%)	4.80	7.20	3.45	6.77	1.87	5.27

PLD: poultry litter dose; TILLERING: tillering; FAULTS: faults in the lines; LSD: lower stem diameter; MSD: middle stem diameter; USD: upper stem diameter; Height: sugarcane height; AWS: average weight of the stem; AWL: average weight of the line; PROD: productivity; TRS: total recoverable sugars; RSS: reducing sugars in sugarcane; BRIX: percentage of dissolved soluble solids; FIBER: sugarcane fiber; PURITY: purity of sugar cane; POL: percentage of oligosaccharides in the sugar produced; CV (%): percentage of coefficient of variation.

The highest tillerings (TILLERING) were found for doses of 2, 4 and 8 t ha ^-1^ and the lowest for doses of 0 and 6 t ha^−1^.

**Figure 3:**
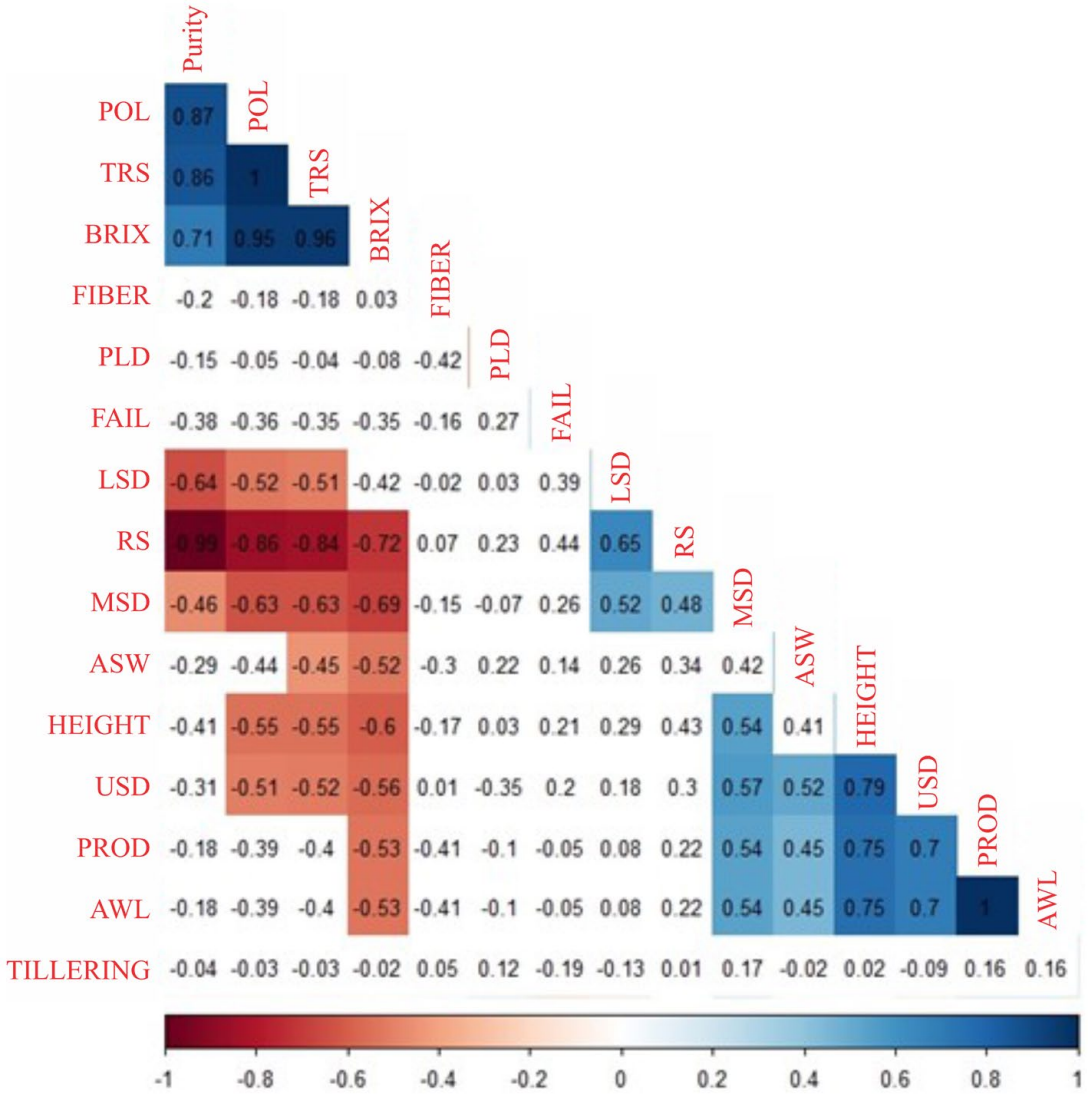
Correlation between variables of sugarcane submitted to different doses of poultry litter. PLD: poultry litter dose; TILLERING: tillering; FAULTS: faults in the lines; LSD: lower stem diameter; MSD: middle stem diameter; USD: upper stem diameter; Height: sugarcane height; AWS: average weight of the stem; AWL: average weight of the line; PROD: productivity; TRS: total recoverable sugars; RSS: reducing sugars in sugarcane; BRIX: percentage of dissolved soluble solids; FIBER: sugarcane fiber; PURITY: purity of sugar cane; POL: percentage of oligosaccharides in the sugar produced; CV (%): percentage of coefficient of variation.

The correlation analysis verified the formation of three groups of variables (Fig. 3): 1. PURITY, total sugar recovered (TRS), percentage of oligosaccharides in the sugar produced (Pol) and percentage of dissolved soluble solids (BRIX); 2. lower stem diameter (IND), reducing sugars (RS), average stem diameter (MSD), average stem weight (ASW), sugarcane height (HEIGHT), upper stem diameter (USD), productivity (PROD), and average line weight (AWL); 3. FIBER, poultry litter dose (PLD), FAULTS and tillering (TILLERING).

The variables within group 1 and within group 2 showed positive correlations, whereas between these groups, the correlations were negative. The variables in group 3 did not show significant correlations with the other variables (Fig. 3).

It was verified by the correlation test that the variables PURE, Pol, TRS and Brix presented high and positive correlations among themselves (r > 0.70), and the same was observed between the variables HEIGHT, USD, PROD and AWL. RS showed a high and negative correlation with PURITY, Pol, TRS and BRIX (r < − 0.70). Correlations below |0.44| were all nonsignificant (Fig. 3).

The factorial multivariate analysis showed that the first four factors had eigenvalues greater than one, jointly explaining 100% of the data variation (Table 5). The first, second, third and fourth factors explained 54.63, 24.28, 12.78 and 8.29%, respectively.

**Table 5:** Classification of factors, eigenvalues, percentages of variance and accumulated percentages of generated variance.

Factors	Eigenvalues	percentage variance	Accumulated percentage
I	8.20 × 10	5.46 × 10^2^	54.6
II	3.64 × 10	2.43 × 10^2^	78.9
II	1.92 × 10	1.28 × 10^2^	91.7
IV	1.24 × 10	8.29 × 10	100.0
V	8.53 × 10^−15^	5.69 × 10^−14^	100.0
VI	4.79 × 10^−15^	3.19 × 10^−14^	100.0
VII	3.92 × 10^−15^	2.61 × 10^−14^	100.0
VIII	2.35 × 10^−15^	1.57 × 10^−14^	100.0
IX	2.00 × 10^−15^	1.33 × 10^−14^	100.0
X	1.75 × 10^−15^	1.17 × 10^−14^	100.0
XI	1.65 × 10^−15^	1.10 × 10^−14^	100.0
XII	1.55 × 10^−15^	1.04 × 10^−14^	100.0
XIII	8.18 × 10^−16^	5.46 × 10^−15^	100.0
XIV	6.21 × 10^−16^	4.14 × 10^−15^	100.0
XV	3.07 × 10^−16^	2.05 × 10^−15^	100.0

The highest factor loading observed, regardless of the sign, indicates a greater variation in it. High factor loadings also indicate high correlation with the factor, which can be seen graphically. It was found that nine of the 15 variables had a factorial load above 0.70 with the first factor, showing the importance of the first factor to explain the variables. Three of the fifteen variables had a factorial load above 0.70 in the second factor. It was found that all variables had commonality above 0.65 (Table 6).

**Table 6:** Factor loadings on factors I, II and III, commonality and specific variance of the 15 sugarcane variables subjected to different poultry litter doses.

Variables	LF 1	LF 2	LF 3	LF 4	commonality	variances
TILLERING	^− ^0.6362	^− ^0.1187	0.6662	^− ^0.3703	0.8628	0.1371
FAULTS	0.7792	^− ^0.5306	0.2518	0.2185	0.9522	0.0477
LSD	0.9648	^− ^0.1062	0.0231	0.2391	0.9428	0.0571
MSD	0.7067	0.2267	0.6585	^− ^0.1242	0.9845	0.0154
USD	0.5931	0.5436	0.0513	0.5915	0.6500	0.3499
H EIGHT	0.8682	^− ^0.2349	^− ^0.2026	0.3871	0.8501	0.1498
AWS	0.3631	^− ^0.6074	^− ^0.6779	− 0.1988	0.9604	0.0395
AWL	0.1957	0.8879	− 0.3821	− 0.1646	0.9728	0.0271
PROD	0.1956	0.8879	− 0.3823	− 0.1647	0.9728	0.0271
TRS	− 0.9693	− 0.1067	− 0.1597	0.1532	0.9765	0.0234
RSS	0.8903	− 0.1379	− 0.1471	− 0.4081	0.8334	0.1665
BRIX	− 0.9719	0.0413	− 0.2251	0.0537	0.9971	0.0028
FIBER	− 0.0350	0.9719	0.2253	0.0575	0.9966	0.0033
PURITY	− 0.9049	− 0.0370	0.1048	0.4107	0.8312	0.1687
POL	− 0.9703	− 0.0976	− 0.1454	0.1662	0.9723	0.0276
Variance	8.1954	3.6427	1.9179	8.2900	13.7561	NA
% variance	54.6364	24.2850	12.7861	100.0000	91.7077	NA

LF: load factor; PLD: poultry litter dose; TILLERING: tillering; FAULTS: faults in the lines; LSD: lower stem diameter; MSD: middle stem diameter; USD: upper stem diameter; HEIGHT: sugarcane height; AWS: average weight of the stem; AWL: average weight of the line; PROD: productivity; TRS: total recoverable sugars; RSS: reducing sugars in sugarcane; BRIX: percentage of dissolved soluble solids; FIBER: sugarcane fiber; PURITY: purity of sugar cane; POL: percentage of oligosaccharides in the sugar produced; CV (%): percentage of coefficient of variation.

It was found that the variables LSD, HEIGHT, TRS, RS, BRIX, PURE and POL had the highest factor loadings (> 0.85) in the first factor. AWL, PROD and FIBER were higher with the second factor (> 0.85), TILLERING, MSD and ASW with the third factor (> 0.65), and USD with the fourth factor (> 0.55). (Table 6).

According to the principal component analysis (PCA), factor I explained 54.63% of the total data variation, while factor II explained 24.28% (Table 6 and Fig. 3). This demonstrates that both components are relevant to explain the behavior of variables and treatments. It was also possible to observe that the variables were able to separate the treatments in a coherent way, facilitating the understanding of the results graphically and objectively.

**Figure 4:**
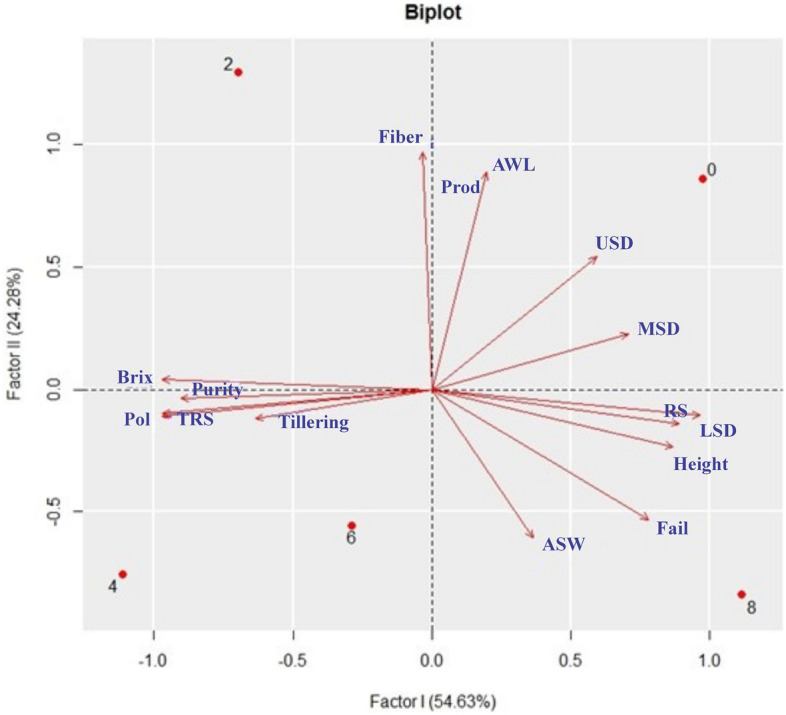
Two-dimensional scatter plots of the factor loading matrix and scores of the 15 variables and treatments.

Figure 4 shows the factorial scores of the first and second factors for the treatments and biometric, production and technological variables evaluated in the present study. Based on the first factor, the greatest similarity between treatments 2, 4 and 6 and between 0 and 8 can be observed. In the second component, greater similarities were observed between treatments 2 and 0 and between treatments 4, 6 and 8.

## Discussions

Analysis of variance is a measure used to calculate how much a set of data can vary, that is, a measure of dispersion. This calculation is necessary to determine the deviation from the mean in relation to a set of analyzed data^13^.

In this sense, when performing the Skott Knott test for sugarcane tillering, the most suitable treatments were 2.4 to 8 t ha^-1^ of poultry litter (PL) (Table 4). The tiller is the basic growth unit of grasses, that is, the period after sprouting and bud development that leads to the emission of culms^13^.

When evaluating the effect of integrated nutrient management on sugarcane growth, productivity and productivity attributes, Arefin et al.^14^ found that the maximum tillers were recorded from the plot treated with poultry litter. The author also reports the importance of using organic sources in sugarcane cultivation, since fertilizer is one of the most expensive inputs for production. There were no significant differences between the other analyzed variables.

Related to TILLERING, greater failures were observed when using the recommendations of 8 t ha^−1^ (Fig. 4). Failures affect the distribution of sugarcane in the planting area since they can promote lower productivity; when there are many failures, replanting is necessary.

Correlation is the existing relationship between phenomena, between mathematical or statistical variables that tend to vary, be associated or occur together in a way not expected just by chance. Thus, it is important to determine the degree of relationship between two phenomena or two variables^15^. In this study, as expected, important positive correlations were found between technological variables and between biometric variables. It was also observed that most of the biometric variables had a negative correlation with the technological variables (Fig. 3).

Another important analysis is the factorial multivariate, which is related to a set of statistical methods that are used in different situations in which the variables are analyzed simultaneously in each sample element. The purpose of this analysis is to simplify or facilitate the interpretation of the results being studied; thus, it helps in understanding the interrelationships of the various varieties that are in any study^16^. This same author explains that a factor is important to explain the variation of the data when its eigenvalue is greater than 1.

When analyzing the factorial load, it is verified that 12 variables are above 0.70 between the first and second factors. Furthermore, the commonality for all variables is above 0.65. The literature recommends that a minimum commonality of 0.5 is considered satisfactory^17^. Commonality is defined as the amount of variance (correlations) of each variable explained by the factors. Thus, the greater potential for explaining that variable by the factor is related to greater commonality^17^.

Regarding stem diameter (LSD, MSD and USD), higher values were found when no PL treatment was used (Fig. 4). In addition to its support function, the sugarcane stalk is very important, as it is rich in sugar. Fibers are also found in its composition. Generally, in the stem, the ratio is 86 to 92% juice to 8 to 14% fibers (water-insoluble material)^18^.

When the dose of 8 t ha was used ^-1^ was used, there was a tendency for sugarcane with greater heights (Fig. 4). Plant height is of great importance for studies with sugarcane, since the production and productivity of this crop depends on its stems, and the tendency is that the greater the plant height is, the greater the number and/or the length of the stem.

Despite not having a significant difference for yield (PROD), when PL was not used and when 2 t ha^−1^ was used, there was a higher yield trend. This means that the increase in poultry litter caused toxicity in sugarcane, since at high doses, it can cause phytotoxicity in plants^19^. PROD is one of the most important factors for the producer, as it shows how much sugarcane is produced tons in one hectare.

In general, FC was beneficial for the biometric and productive parameters of sugarcane. Organic matter improves the physical, chemical and biological properties of the soil. Organic soils have greater microbial activity, which assists in nutrient cycling and consequently releases higher levels of nutrients available to plants. Another important factor is that this type of soil has a greater potential for water retention; thus, it can benefit plants in periods of water deficit^20–24^.

Regarding fiber, the best results were presented with the treatment of 2 t ha^−1^ of CF (Fig. 4 and Table 4). The fibers support and fill the sugarcane. The main components of this compound are lignin (15%), which is responsible for the caloric power of sugarcane, and cellulose (40%) and hemicellulose (35%), both forms of carbohydrates with greater abundance in nature and great reserve potential for sugarcane. The greater the amount of fiber in the cane, the lower the extraction efficiency. The lower the amount of fiber is, the greater the risk of mechanical damage in the harvesting and transport stages, which can favor contamination and some losses in the industry. Another problem is that sugarcane can lodge and even break in the wind, with low fiber contents^25^, ^26^.

About fiber, Consecana^9^ establishes that the ideal value should be between 11 and 13%. Therefore, only the treatment with 2 t ha ^-1^ presented a value in the ideal range; for the other treatments, the percentages were below the reference value. For Matsouoka^27^, the ideal range of sugarcane fibers is 12 ± 2%. According to this author, all fiber percentages are adequate. As mentioned above, above 14%, the extraction efficiency will be low and below 10%, and there may be problems with overturning and damage with the harvest and transport of the cane.

There was a tendency toward higher percentages of reducing sugars (RS) when sugarcane was cultivated with 8 t ha^−1^ of PL and greater purity when cultivated with 4 t ha^−1^ (Table 4). RS is the amount of fructose and glucose present in sugarcane, which can directly affect its purity, which may reflect a lower efficiency in the recovery of sucrose by industry^28,29^. With regard to RS, Ripoli and Ripoli^31^ and Consecana^9^ establish that the appropriate percentage should be less than 0.8%, which means that the values found in this study are adequate.

Purity is the relationship between Brix and the percentage of oligosaccharides (POL). The higher the percentage of this variable, the better the quality of the raw material in terms of sugar recovery^32^. According to Ripoli and Ripoli^31^ and Consecana^9^, the ideal purity value should be greater than 85%; therefore, it is observed that the percentages for sugarcane cultivated with 0 and 8 t ha^-1^ are below the recommended and for the other treatments is as expected by the authors.

The °Brix refers to the percentage of soluble solids dissolved in raw sugarcane juice, that is, in a chemically pure solution of sucrose. That is, the percentage of sucrose, glucose, fructose and other Non sugar compounds such as amino acids, fats, waxes and minerals that are absorbed by sugarcane in its maturation stage^33^. The appropriate °Brix value for the sugar and alcohol industries should be above 18%^9^, ^34^. Table 3 shows that when sugarcane was cultivated at 0 and 8 t ha^−1^, the percentages of °Brix were below the recommended level. There was a tendency toward a higher percentage for the treatment with 4 t ha^−1^ (Table 4).

The percentage of oligosaccharides in the sugar produced (POL) is the mass content of apparent sucrose in the sugarcane crop. Higher levels are better in the sugarcane sector because it correlates directly with the quality final product^33^. Consecana^9^ and Gonçalves et al.^34^ establish that the POL value should be greater than 14%. For all treatments, the percentages were below the recommended levels (Table 4).

The total sugar recovered (TRS) indicates the total amount of sugars available from the cane (fructose, sucrose and glucose) and is one of the factors most desired by the industry. In general, the percentage of sugars in this crop is in the range of 13 to 17.5%^35^, ^36^. With regard to TRS, for the 2021/22 harvest, Goiás had an average of 147.6 kg t^−1^, while the national average was 141.6 kg t^−1^^1^. All values found were below the average for the state of Goiás and for Brazil; however, there was a greater tendency for higher TRS for sugarcane cultivated at 4 t ha ^-1^.

## Conclusions

It can be seen from this test that the insertion of poultry litter did not interfere significantly in most biometric, productive and technological variables. Therefore, it can also be observed that there was a statistical trend of better results when it was cultivated with 4 t ha^-1^ of poultry litter.

Such trend can be attributed to the benefits arising from the addition of organic matter to the soil and its influence on physical factors such as improving the structure and stability of soil aggregates and increasing the water retention capacity, biological factors by stimulating microbial activity, and chemical factors by influencing the availability of nutrients through their mineralization.

## Plant guideline statement

Experimental research and field studies on cultivated plants, including the collection of plant material, comply with the required institutional, national and international guidelines and legislation. The experimental area used belongs to Denusa Sugar and Alcohol Plant Destilaria Nova União where the study was carried out.

## Data Availability

The datasets used and analysed during the current study is available from the corresponding author on request.
